# Associated factors of diabetic retinopathy in patients that referred to teaching hospitals in Babol 

**Published:** 2015

**Authors:** Seyed Ahmad Rasoulinejad, Karimollah Hajian-Tilaki, Elnaz Mehdipour

**Affiliations:** 1 Department of Ophthalmology, Ayatollah Rouhani Hospital, Babol University of Medical Sciences, Babol, Iran.; 2 Department of Biostatistics and Epidemiology, Babol University of Medical Sciences, Babol, Iran.; 3 Babol University of Medical Sciences, Babol, Iran.

**Keywords:** Diabetic retinopathy, Diabetes mellitus, Risk factors

## Abstract

**Background:**

Information regarding the frequency and severity of eye involvement in diabetic patients and its risk factors can be useful for prevention and treatment. This study aimed to determine the prevalence of diabetic retinopathy and its associated risk factors in diabetic patients who referred to teaching hospitals in Babol, Mazandaran.

**Methods:**

In this study a total of 1562 patients with the definite diagnosis of diabetes mellitus were chosen and referred to the eye clinic of Shahid Beheshti and Ayatollah Rouhani Hospitals of Babol during 2006-2010. Information about age, duration of diabetes, hypertension family history of diabetes, history of other organs involvement, BMI and laboratory findings were recorded. Retinopathy was classified according to early treatment of diabetic retinopathy study. Data were collected and analyzed.

**Results:**

Of the 1562 patients, 357 (22.9%) were males and 1205 (77.1%) were females. The mean age was 54.6±10.6 years, diabetic retinopathy was found in 990 (64.1%) patients, the frequency of non-proliferative diabetic retinopathy was 37.3% and proliferative was 26.8%.Age, duration of diabetes, FBS, HbA1C, BUN, Cr, Hb were significantly different between the patient’s with diabetic retinopathy and no diabetic retinopathy (p<0.05). No relationship was found between smoking, gender, hypertension and serum lipid profile.

**Conclusion:**

Poor diabetes control, anemia and nephropathy were the most associated factors of diabetic retinopathy but hypertension BMI smoking, dyslipidemia and gender showed no association.

Diabetic retinopathy is a retinal vascular abnormality; due to chronic hyperglycemia which is one of the four leading causes of blindness in the world and the most common cause of new cases of blindness in adults 20 to 74 years in Western societies (1). Diabetic retinopathy is classified into early stage, nonproliferative diabetic retinopathy (NPDR), and more advanced stage, proliferative diabetic retinopathy (PDR), Retinopathy was evaluated according to the classification of ETDRS (early treatment of diabetic retinopathy study) (2). Blindness happens primarily as a result of advanced retinopathy (3). The prevalence of retinopathy in diabetic population increases with age and duration of diabetes. Approximately 60-90% of patients in both types of diabetes have diabetic retinopathy over 20 years (4-6). Considering the high prevalence of diabetes and diabetic retinopathy, studies were done to evaluate the incidence of diabetic retinopathy and its association with age, sex, duration of diabetes, blood pressure, serum lipid profile, blood glucose control and body mass index (7-9).

In some studies, duration of diabetes and the level of blood glucose control were the best predictors for the development of retinopathy (5, 6, 10). The purpose of this study was to determine the prevalence of diabetic retinopathy and its associated risk factors in diabetic patients that referred to teaching hospitals in Babol, Mazandaran.

## Methods

This cross-sectional study involved 1562 patients with definitive diagnosis of diabetes mellitus that referred to the eye clinic of Shahid Beheshti and Ayatollah Rouhani Hospitals during 2006-2010. Evaluation of retina was made by Slit-Lamp biomicroscopy of the posterior pole using contact lens after dilation of pupil with tropicamide 1% eye drop. The study information consisting of age, sex, duration of diabetes, hypertension, heart disease, nephropathy, neuropathy, insulin treatment, Hb, HbA1C, FBS, BS 2hpp, BUN, creatinine, cholesterol, TG, HDL, LDL, and VLDL were measured and recorded. Data were analyzed by SPSS software (Version 18). Chi-square test was used for categorical data and ANOVA for quantitative data. P<0.05 was considered significant.

## Results

This study included 1562 patients, 1205 (77.1%) women and 357 (22.9%) men. The mean age was 54.6±10.6 years. The mean duration of diabetes was 9.6±7 years in men and 10.9±7.4 years in women. Diabetic retinopathy was observed in 990 (64.1%) patients, in which 576 (37.3%) had non-proliferative (NPDR) and 414 (26.8%) had proliferative diabetic retinopathy (PDR).

The mean BMI was 27.84±3.9Kg/m²; the mean BMI among women (28.03±4.1) was significantly higher than men (27.2±3.6) (P<0.001), family history of diabetes was positive in 299 (83.7%) of men and 1067 (88.5%) of women with significant difference between the sexes (P<0.05). Diabetes control with insulin was in 333 (21%) patients, 62 (17%) men and 271 (22.4%) women; the difference between the sexes in using insulin was significant (p<0.05). Frequency and severity of retinopathy according to potential risk factors are listed in table 1.

Table 1 showed that heart disease, nephropathy, neuropathy, insulin treatment and family history of diabetes were associated with severity of retinopathy. There was no significant relationship between retinopathy and gender, history of hypertension and smoking (table 1). 

**Table1:** Frequency and severity of diabetic retinopathy according to potential risk factors

**P-value**	**Retinopathy** **N (%)**	**Normal** **N (%)**	**Characteristics **
0.96	231 (22.9) 778 (77.1)	126 (22.8) 427 (77.2)	**Gender** male Female
0.005	109 (10.8) 900 (89.2)	87 (15.7) 466 (84.3)	**Family History** No Yes
0.001	717 (71.1) 292 (28.9)	512 (92.6) 41 (7.4)	**Insulin Treatment ** No Yes
0.2	572 (56.7) 437 (43.3)	330 (59.7) 223 (40.3)	**Hypertension** No Yes
0.003	859 (85.1) 150 (14.9)	500 (90.4) 53 (9.6)	**Heart disease** No Yes
0.001	937 (92.9) 72 (7.1)	547 (98.9) 6 (1.1)	**Neuropathy** No Yes
0.001	988 (97.9) 21 (2.1)	543 (98.2) 10 (1.8)	**Nephropathy** No Yes
0.9	929 (92.1) 80 (7.9)	552 (94.4) 31 (5.6)	**Smoking** No YES

Two hundred three (12.9%) patients had heart disease, 146 females and 57 males, the difference between the sexes was not significant. 7(0.4%) patients had nephropathy in which 3 patients had renal transplantion. 78 (5%) patients had neuropathic complications in which 32 patients had diabetic foot in which 9 cases resulted in amputation, 7 patients had sixth nerve paralysis. The difference between genders was not significant (p>0.05). Hypertension was present in 660 (42.2%) patients, 546 women and 114 men and the prevalence was significantly higher in women (p<0.001). Mean±SD, 95% CI, p-value and quantitative variables are presented in table 2. As shown in table 2, the relationship between the severity of retinopathy and age, duration of diabetes, BMI, Hb, HbA1c, FBS, BS 2hpp, BUN and creatinine was significant (P<0.05). However, the relationship between cholesterol, TG, HDL, LDL and VLDL with retinopathy was not significant (p>0.05).

**Table 2:** Mean±SD quantitative variables of diabetic retinopathy

**P-value**	**Retinopathy** **Mean±SD**	**Normal** **Mean±SD**	**Characteristics **
0.001	55.6±9.8	52.8±11.9	Age (year)
0.001	13.1±7.3	6.1±5.5	Duration of diabetes (year)
0.001	27.6±4.02	28.2±14.1	BMI (Kg/m^2^)
0.001	12.5±1.7	12.9±1.6	Hemoglobin (mg/dl)
0.001	9.3±2.0	8.1±1.9	HbA1c (%)
0.001	204±76.3	175±64.3	FBS (mg/dl)
0.001	85±92	245±289	Bs2hpp (mg/dl)
0.008	28.9±17.8	23.7±15.4	BUN (mg/dl)
0.002	0.98±0.54	0.88±0.34	Creatinine (mg/dl)
0.2	204±56.5	200±59.1	Cholesterol (mg/dl)
0.1	205±127	205±126	TG (mg/dl)
0.9	44.2±14.3	44.4±16.2	HDL (mg/dl)
0.16	115.6±39.9	112.3±38.1	LDL (Mg/dl)
0. 7	4.09±29.3	39.6±27.2	VLDL (mg/dl)

## Discussion

In this study, the overall prevalence of diabetic retinopathy was 64.1%, non-proliferative 37.3% and proliferative 26.8%. The prevalence of diabetic retinopathy in different studies was reported from 26 to 50 percent (11). As seen in studies conducted in different countries, there is a remarkable difference in the prevalence of non-proliferative diabetic retinopathy from 24 to 70 percent and proliferative diabetic retinopathy from 4.5 to 22 percent (12). These differences may be related to quality of life and geographical conditions. In a study conducted by D.P.Liu et al. in China, the prevalence of diabetic retinopathy was 27.3% (13).

The study of Rafati et al. in Tehran on 700 patients with diabetes have been diagnosed recently, the prevalence of retinopathy was reported 37.9% (14); the reason for the difference between this study and the present study was to investigate only the people who have recently suffered from diabetes.In the study of Aqadoust et al. in Iran, there was 36% retinopathy in diabetic patients (11). In the study of Malekmadani et al., 77.4% of diabetic patients had retinopathy (15) and 51.1% in the study of Mazarei (16), which is also statistically similar to our study. In this study, the several risk factors associated with retinopathy were evaluated and showed that many factors are associated with retinopathy including :age, duration of diabetes, history of ischemic heart disease, nephropathy, neuropathy, insulin consumption, family history of diabetes, body mass index (BMI), serum hemoglobin level, HbA1c, FBS, BS2hpp, BUN and creatinine. Factors such as gender, history of hypertension, smoking, cholesterol, triglyceride, HDL, LDL and VLDL levels did not have a significant relationship with retinopathy.

In this study, the age of patients had a significant relationship with retinopathy, which is consistent with the studies of Mazarei and Rouhipour (16, 17); it can be related to the increasing duration of diabetes and other risk factors with aging. The relationship between duration of diabetes with the prevalence of retinopathy was significant. It also had been shown in other studies (11-15, 18-20), that after 14 years of diabetes, the prevalence of diabetic retinopathy was reported 5 to 25%, while in less than 14 years, this probability was 3.5% (11).

In this study, the patients with retinopathy had poor glycemic control than the patients without retinopathy, and the high levels of fasting blood glucose was associated with higher grades of retinopathy, thus, this rate in patients without retinopathy was 175±64.3mg/dl, NPDR 204±73 mg/dl, and PDR 213±82 mg/dl, (p<0.001). Also, BS2hpp and HbA1C had significant relationship with retinopathy (p<0.001), that is consistent with other studies (11, 13, 16, 18, 21) 

Rema et al.’s study have shown that the duration of diabetes, severity of hyperglycemia and hyperlipidemia are the most important risk factors for progression of diabetic retinopathy, while hypertension has no role (19). Another study conducted by Liu et al. showed that factors such as age, sex, duration of diabetes, BMI, systolic blood pressure, HbA1C and insulin consumption have relationship with the severity of diabetic retinopathy, while the age of onset of diabetes, high diastolic blood pressure, high blood cholesterol, triglycerides, and smoking had no significant relationship with retinopathy (13).

The study of Pradeepa et al. showed that the duration of diabetes, the level of HbA1c, insulin treatment and males are significantly associated with the severity of retinopathy (18); Malek Madani’s study found that the factors related to retinopathy include high blood pressure, increased serum creatinine, duration of diabetes, nephropathy and diabetic foot (15). Another finding of this study was the examination of more women (1205) than men (357) with diabetes, perhaps due to the lack of sufficient opportunities for employment in men and the importance of their health status in women. However, the prevalence of retinopathy was not different in two sexes, the study of Pradeepa, Rafati, Rouhipour and Aqadoust showed that the incidence and severity of retinopathy has a significant relationship with male sex (11, 12, 14, 17).

In this study, the effect of blood pressure on the prevalence of retinopathy was not significant, Studies had different results, Malekmadani and Rouhipour showed that the incidence of retinopathy was associated with high blood pressure; Akbarzadeh in a study in Hamedan showed hypertension with 54.6% frequency had the highest frequency with retinopathy. Manaviat et al.’study also showed that the risk of retinopathy in patients with high blood pressure was 1.37 times higher than those without high blood pressure (P<0.05);. Some studies, including the study of Rema and Aqadoust did not find any relationship between these two factors.

In our study, insulin treatment is associated with increased frequency and severity of retinopathy, In the study of Batram and Pradeepa and also a study conducted in the USA by Broadbent et al., the prevalence of retinopathy in patients using insulin was higher (18, 22, 23), In Rafati et al.’s study, the risk of retinopathy in those who used insulin was 1.55 times higher than the patients that used oral drugs (14), These findings show that the patients who are treated with insulin have usually more severe glycemic disorder or do not respond to oral therapy, therefore, the obtained results are more justified and partly expected based on the severity of retinopathy in patients treated with insulin. In our study, gender, hypertension, smoking, level of cholesterol, triglycerides, HDL, LDL and VLDL had no significant relationship with retinopathy.

In the present study, the BMI was negatively but the family history was positively associated with retinopathy. In some studies, BMI was reported as a risk factor for developing retinopathy (13, 17), while some studies failed to show a significant relationship between the two (21). In the study of Khalilian et al., there was no relationship between retinopathy and family history of diabetes (24). Increased levels of BUN and creatinine are related with retinopathy especially in the study of Aqadoust and MalekMadani that is consistent with our results. Based on the results of the present study, a history of heart disease, neuropathy and nephropathy is significantly associated with seventy of retinopathy. Nephropathy and diabetic foot in the study of Malekmadani, and chronic renal failure and ischemic heart disease in Akbarzadeh et al.’s study were also associated with diabetic retinopathy (15, 25), but Aqadoust did not report ischemic heart disease as the risk factor of retinopathy (11). Lesaffre et al. reported that the duration of diabetes, HbA1C, diastolic blood pressure, proteinuria, sex, macular edema, and refractive errors were the factors influencing the severity of retinopathy (11, 17).

In conclusion, the results of this study demonstrated that factors like age, duration of diabetes, poor diabetes control, nephropathy and anemia were significant associated risk factors of diabetic retinopathy but hypertension, smoking, gender and dyslipidemia were not associated. This issue requires further studies. 

## Notes

**Funding: **This study is a thesis project of Elnaz Mehdipour (No: 1192).
